# Flexion reminder device to discourage recurrent posterior dislocation of a total hip replacement: a case report

**DOI:** 10.1186/1752-1947-2-250

**Published:** 2008-07-25

**Authors:** King Wong, Manoj Sivan, Gordon Matthews

**Affiliations:** 1 Department of Orthopaedics, Wycombe General Hospital, High Wycombe, Buckinghamshire, UK

## Abstract

**Introduction:**

Recurrent dislocation of a total hip replacement prosthesis is a frustrating complication for both the surgeon and the patient. For positional dislocations with no indications for revision surgery, the current best treatment is physiotherapy, the use of abduction braces and avoidance of unsafe hip positions. Abduction braces can be cumbersome and have poor compliance. We report the successful use of a new lightweight flexion reminder device that can be used to treat people with this condition.

**Case presentation:**

A 64-year-old British woman experienced recurrent positional posterior dislocation after primary hip replacement, particularly when involved in activities involving unsafe flexion of the operated hip. She disliked using an abduction brace and hence was given a simple 'flexion reminder device' that could be strapped to the thigh. Beyond the safe flexion limit, the padded top end of the device hitched against the groin crease and reminded her not to flex further, to avoid dislocation. She experienced no discomfort in wearing the device continuously throughout the day and was very satisfied. She has had no further dislocations in the 2 years since she began using it.

**Conclusion:**

In cases of arthroplasty dislocation caused mainly by an unsafe hip position, and with no indication for revision surgery, this new lightweight and easily worn flexion reminder device may be a good option for avoiding such positional dislocations, particularly those caused by unsafe flexion.

## Introduction

Total hip arthroplasty is an extremely successful operation for relieving pain and restoring function. Dislocation of the prosthesis is one of the most disappointing potential postoperative complications. The incidence of dislocation has been reported to vary from 1% to 7%, depending on the follow-up duration [1,2].

The main causes of recurrent dislocation are component malposition, soft-tissue imbalance or positional reasons [3]. Revision surgery is recommended only when the cause of instability can be identified, such as component malposition or soft-tissue imbalance. For those with positional dislocations and no other obvious identifiable cause, the current best treatment involves educating the patient about unsafe hip positions, using abduction braces and physiotherapy to restore the hip musculature around the prosthesis.

However, the benefits of using abduction braces to prevent dislocation remain controversial [4]. The braces are costly, bulky and disliked by patients, resulting in poor compliance. We describe a new, simple device, developed based on a patient's idea, which has helped to prevent further dislocation.

## Case presentation

**Figure 1:**
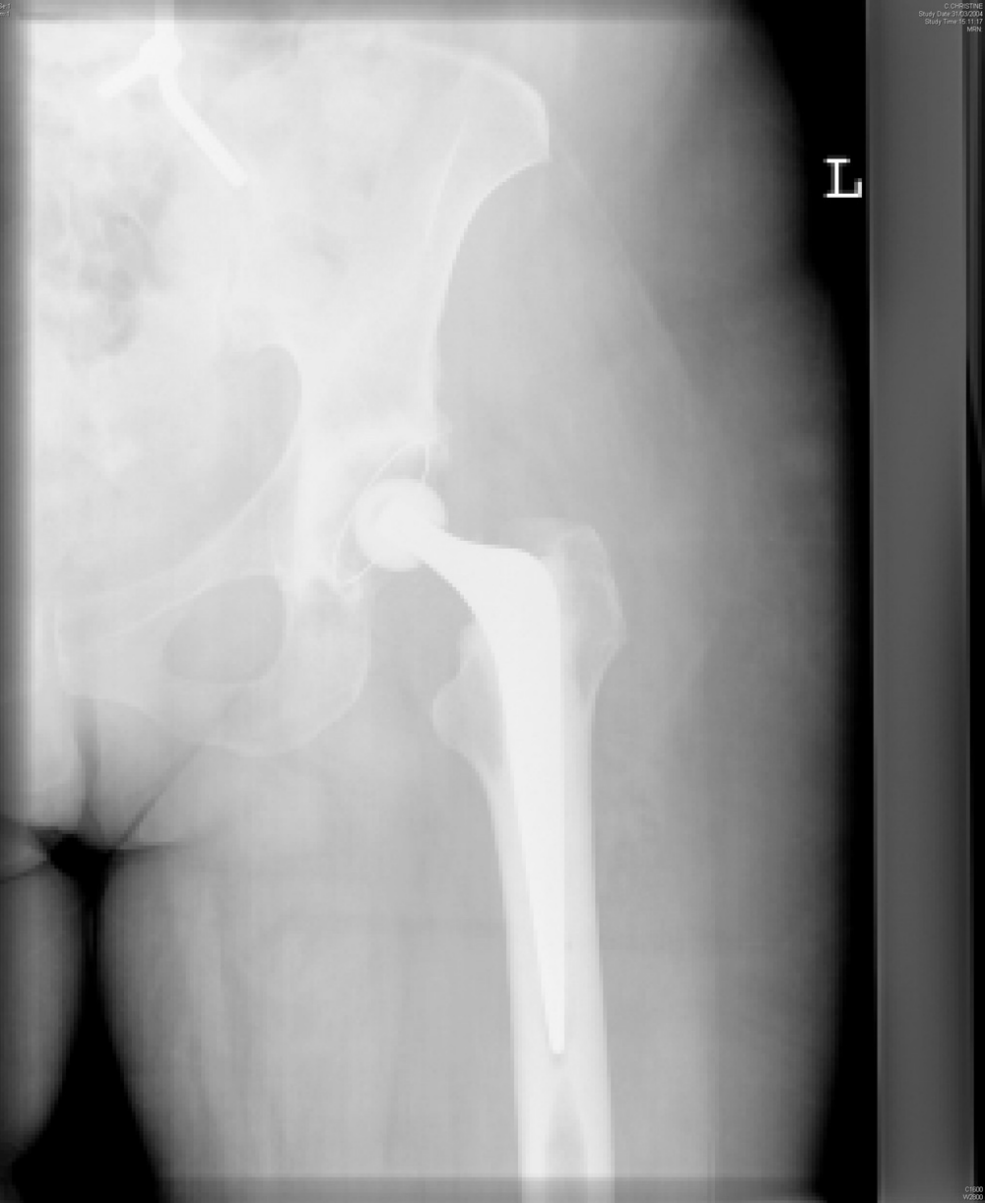
Post-reduction anterior-posterior radiograph of the hip arthroplasty.

**Figure 2:**
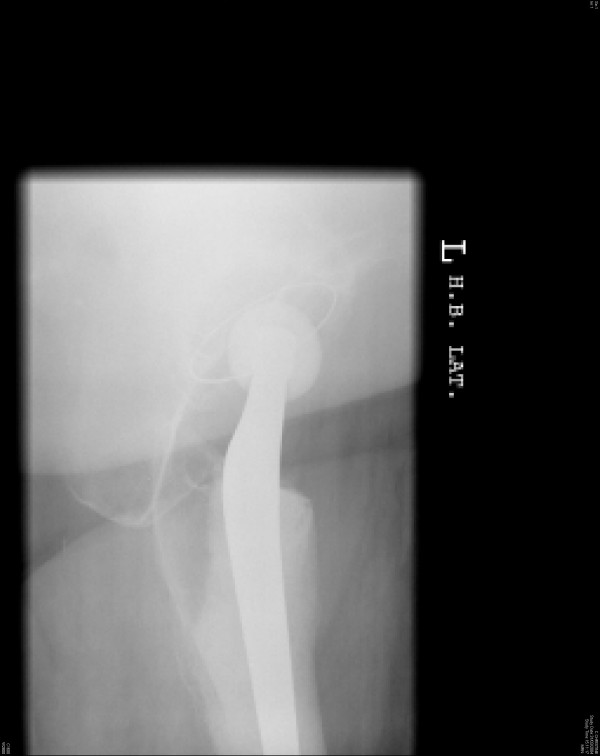
Lateral view of the relocated hip arthroplasty.

A 64-year-old fit and healthy woman had a left cemented total hip replacement using an anterolateral approach for primary osteoarthritis of the hip. Six weeks later, she bent down to pick up an object from the floor and dislocated her operated hip. Relocation of the hip was performed under general anaesthesia. At post-reduction examination under the effect of anaesthesia, the hip was stable within the safe range of movements. X-rays of the relocated hip arthroplasty did not show any component malposition (Figures 1 and 2). She received comprehensive physiotherapy and advice on avoiding unsafe positions.

She eventually had three more dislocations over a period of 2 years (when getting into the bath tub and sitting in a low sofa). All four dislocations required hospital admission and reduction under general anaesthesia. The patient admitted that the precipitating cause for each dislocation was an unguarded flexion of the hip. An abduction brace was prescribed but she could not tolerate wearing it all the time. She suggested having a simple device strapped to her thigh which would physically remind her when she should not flex beyond a limit.

**Figure 3:**
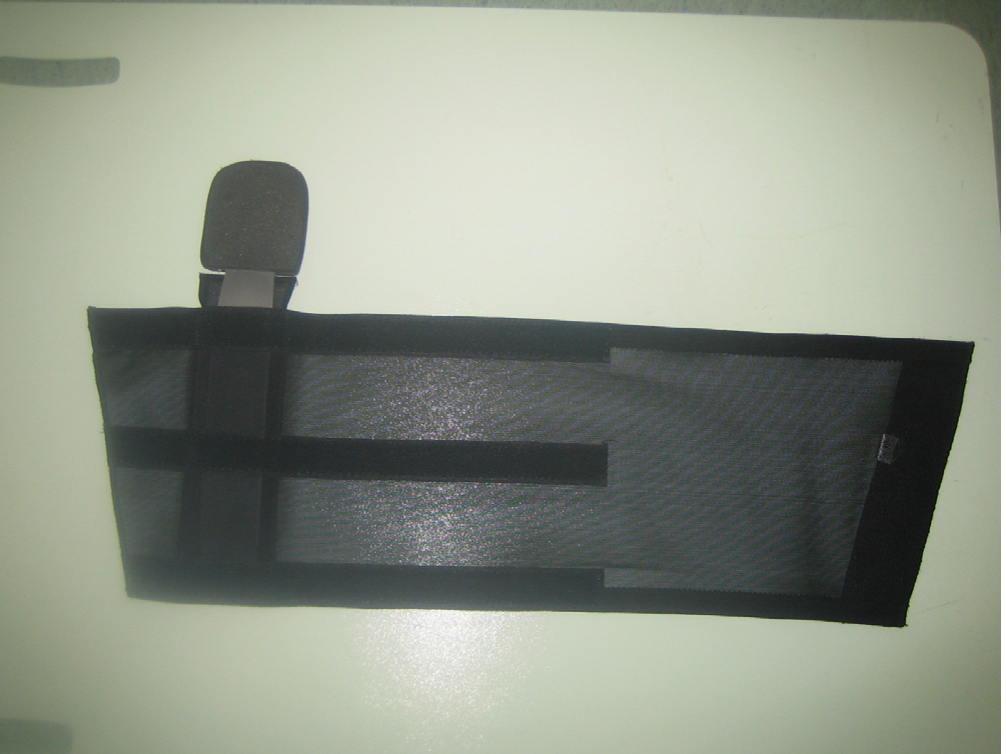
The orthosis (called a 'flexion reminder device') has a simple padded plastic device within an elastic strap with a Velcro fastening.

**Figure 4:**
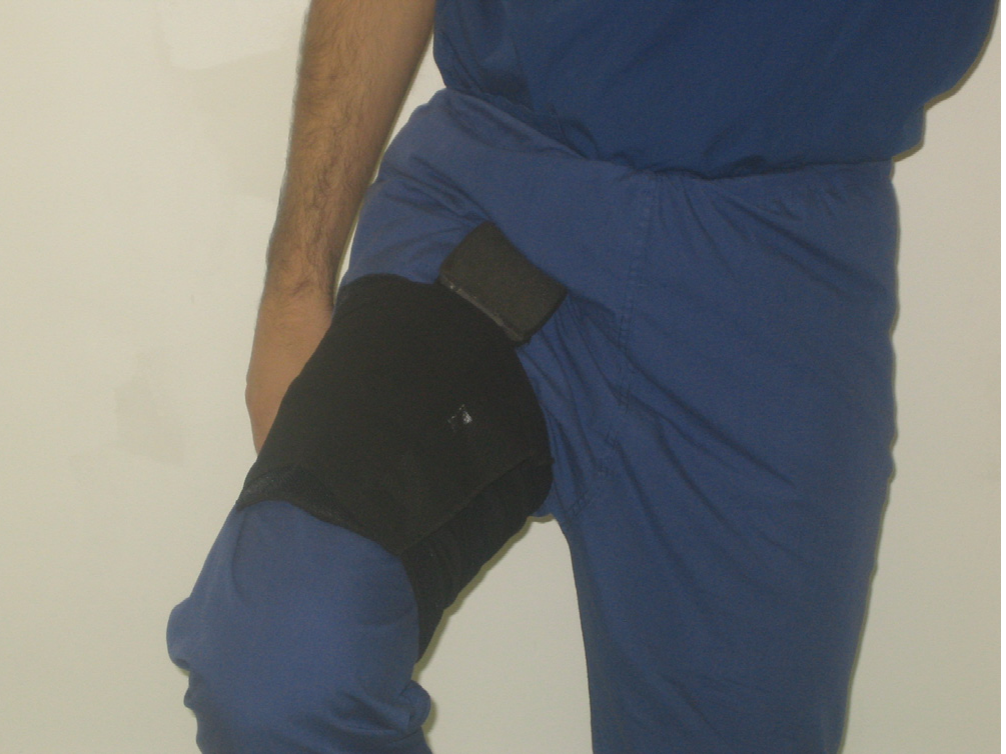
The padded portion of the device hitches against the groin when the hip is flexed beyond 70° and acts as a reminder.

Based on this suggestion, we devised a simple padded plastic device which was strapped to the upper thigh using an elastic strap with a Velcro fastening (Figure 3). Beyond about 70° of flexion, the padded portion of the device hitches against the groin crease and reminds the patient not to flex further (Figure 4).

The patient wore the device under her normal clothing. She wore it continuously, including sleeping with it in place. She wore it to get into the bath and removed it temporarily while washing. She has been using this device for the 2 years since the last dislocation and has had no further dislocations. She is very comfortable using it and has now learnt her limitations in terms of hip movements and lifestyle. She is now slowly weaning off its continuous use.

## Discussion

Recurrent dislocation is a frustrating complication for both the patient and the surgeon. Although the cause is multifactorial, the three main causes for recurrent dislocation are component malposition, soft-tissue deficiency and positional reasons [3]. Component malposition, when identified, can be effectively corrected with revision of the malpositioned component [3,5]. Soft tissue imbalance can be effectively treated with trochanteric transplantation, adjusting the neck length or with constrained acetabular liners [3,5,6]. However, revision surgery is challenging and problems related to further dislocations, premature wear, increased radiolucency, fractures and dislodgement of the liners remain major concerns [7,8].

In patients with no component malposition or soft-tissue imbalance (mainly positional dislocation) and those who refuse or are unfit for revision surgery, the best management strategy is to protect the hip and re-educate the patient about unsafe positions where the hip is likely to dislocate.

Abduction braces maintain the hip in a theoretically safe position and have been used widely by patients who suffer recurrent dislocations. Their effectiveness, however, remains controversial. A recent study of 149 patients with no malpositioned components showed no significant reduction in redislocation rate [4]. However, this was a retrospective study which included patients operated on by different surgeons, where soft-tissue imbalance was not considered as an exclusion criteria and where compliance with the brace was not recorded.

Surgeons advocating abduction braces expect their patients to wear them continuously, except while bathing. However, the braces are bulky and patients dislike them. Patient compliance with these braces is definitely questionable. The rationale of using braces is to prevent the hip from moving into unsafe positions of extreme flexion and adduction.

Our simple 'Flexion Reminder device' acts as a physical reminder when the hip is flexed beyond safe limits (Figure 4). Beyond 70° of flexion, the padded top portion of the device hitches against the groin crease and reminds the patient not to flex further. This device can be worn either under or over clothing. It can be worn continuously for the whole day, including while sleeping. It can be removed while bathing or the sponge top of the plastic device can be dried easily if worn while bathing. The device can be worn while sitting in high chairs. As long as there is no flexion beyond 70°, the device is not uncomfortable while sitting. The device is very secure when worn and does not become loose, even when worn continuously for a whole day. The device is lightweight and cheap to manufacture.

In addition to preventing unsafe flexion, it could be argued that the device could potentially be used to prevent unsafe adduction and internal rotation, by strapping the device to the thigh in a more medial position to the standard (Figure 4 demonstrates the standard position to prevent unsafe flexion alone).

We plan to recruit suitable patients with primary or revision hip arthroplasty who are experiencing recurrent posterior dislocation with no indication for revision surgery. This device could be tried with patients who are non-compliant with abduction braces.

## Conclusion

In cases of arthroplasty dislocation mainly due to unsafe hip positions and with no indication for revision surgery, this new lightweight and easily worn 'Flexion Reminder' device may be a good option in avoiding such positional dislocations, particularly those caused by unsafe flexion.

## Competing interests

The authors declare that they have no competing interests.

## Authors' contributions

KW identified the problem, arranged for the device to be tried by the patient and wrote the paper. MS followed up the patient, edited the paper and shot the device demonstration photographs for the paper. GM was the main surgeon, arranged for the device to be made and edited the paper. All authors have read and approved the final manuscript. GM should be contacted for any further enquiries about the device

## Consent

Written informed consent was obtained from the patient for publication of this case report and accompanying images. A copy of the written consent is available for review by the Editor-in-Chief of this journal.
